# Invasion Success in a Marginal Habitat: An Experimental Test of Competitive Ability and Drought Tolerance in *Chromolaena odorata*

**DOI:** 10.1371/journal.pone.0068274

**Published:** 2013-08-01

**Authors:** Mariska te Beest, Kelly Elschot, Han Olff, Rampal S. Etienne

**Affiliations:** 1 Community and Conservation Ecology Group, University of Groningen, Groningen, The Netherlands; 2 Department of Ecology and Environmental Science, Umeå University, Umeå, Sweden; University of Leipzig, Germany

## Abstract

Climatic niche models based on native-range climatic data accurately predict invasive-range distributions in the majority of species. However, these models often do not account for ecological and evolutionary processes, which limit the ability to predict future range expansion. This might be particularly problematic in the case of invaders that occupy environments that would be considered marginal relative to the climatic niche in the native range of the species. Here, we assess the potential for future range expansion in the shrub *Chromolaena odorata* that is currently invading mesic savannas (>650 mm MAP) in South Africa that are colder and drier than most habitats in its native range. In a greenhouse experiment we tested whether its current distribution in South Africa can be explained by increased competitive ability and/or differentiation in drought tolerance relative to the native population. We compared aboveground biomass, biomass allocation, water use efficiency and relative yields of native and invasive *C. odorata* and the resident grass *Panicum maximum* in wet and dry conditions. Surprisingly, we found little differentiation between ranges. Invasive *C. odorata* showed no increased competitive ability or superior drought tolerance compared to native *C. odorata*. Moreover we found that *P. maximum* was a better competitor than either native or invasive *C. odorata*. These results imply that *C. odorata* is unlikely to expand its future range towards more extreme, drier, habitats beyond the limits of its current climatic niche and that the species’ invasiveness most likely depends on superior light interception when temporarily released from competition by disturbance. Our study highlights the fact that species can successfully invade habitats that are at the extreme end of their ranges and thereby contributes towards a better understanding of range expansion during species invasions.

## Introduction

Understanding the mechanisms that determine the success of invasive species is of fundamental importance to limit their negative impact on biodiversity and ecosystem functioning [1]. A common rule-of-thumb in invasion biology is that for a species to be able to establish, persist and spread in a new environment, the set of ecological conditions in the new environment must approximately match the ecological conditions in their native environment, a phenomenon known as niche conservatism [2]. This view is used widely to predict the distribution of invasive species in climate matching models [3], [4] and ecological niche modeling [5], [6]. However, such models often lack a clear understanding of underlying ecological and evolutionary processes, which limits the ability to predict future range expansion of invasive species [7].

For plants, determinants of range limits include abiotic factors such as climate (temperature and precipitation) or soil conditions, biotic factors such as competition, herbivory or pathogens, or simply time for dispersal [8]. In addition to ecological factors, evolutionary processes also play a role in determining species ranges [9]. For example, maladaptive gene flow, low genetic variation and/or genetic correlations may prevent adaptation to marginal habitats [10]. In many cases introduced species can overcome these constraints on range margins, for example through enemy or competitor release, long-distance dispersal, release from gene flow from the native range and/or admixture of multiple introduced populations [11].

These factors may enable introduced plants to expand their non-native ranges and potentially become invasive. Generally, the underlying assumption is that invasive plants will only become successful and dominant where habitat conditions are optimal. However, what happens when species successfully invade environments that would be considered marginal relative to the climatic niche in the native range of the species? This could occur through (1) post-introduction adaptation to the local environment, or (2) the introduction of either pre-adapted genotypes or genotypes with a broad environmental tolerance. Alternatively, (3) the conditions might actually be marginal in the native range only, for example due to the presence of particular natural enemies or other biotic constraints. These three different scenarios lead to very different predictions for the risk of future range expansion of the invasive species. In scenarios 1 and 3 future ranges may expand into habitat that was previously considered unsuitable, which may be difficult to predict, whereas in scenario 2, accurate predictions can be made based on the species’ native climatic niche. Differentiating between these processes underlying invasion success will enable us to gain more insight in the risk of future range expansion of exotic invaders and may allow us to adjust our control strategies accordingly.

**Figure 1:**
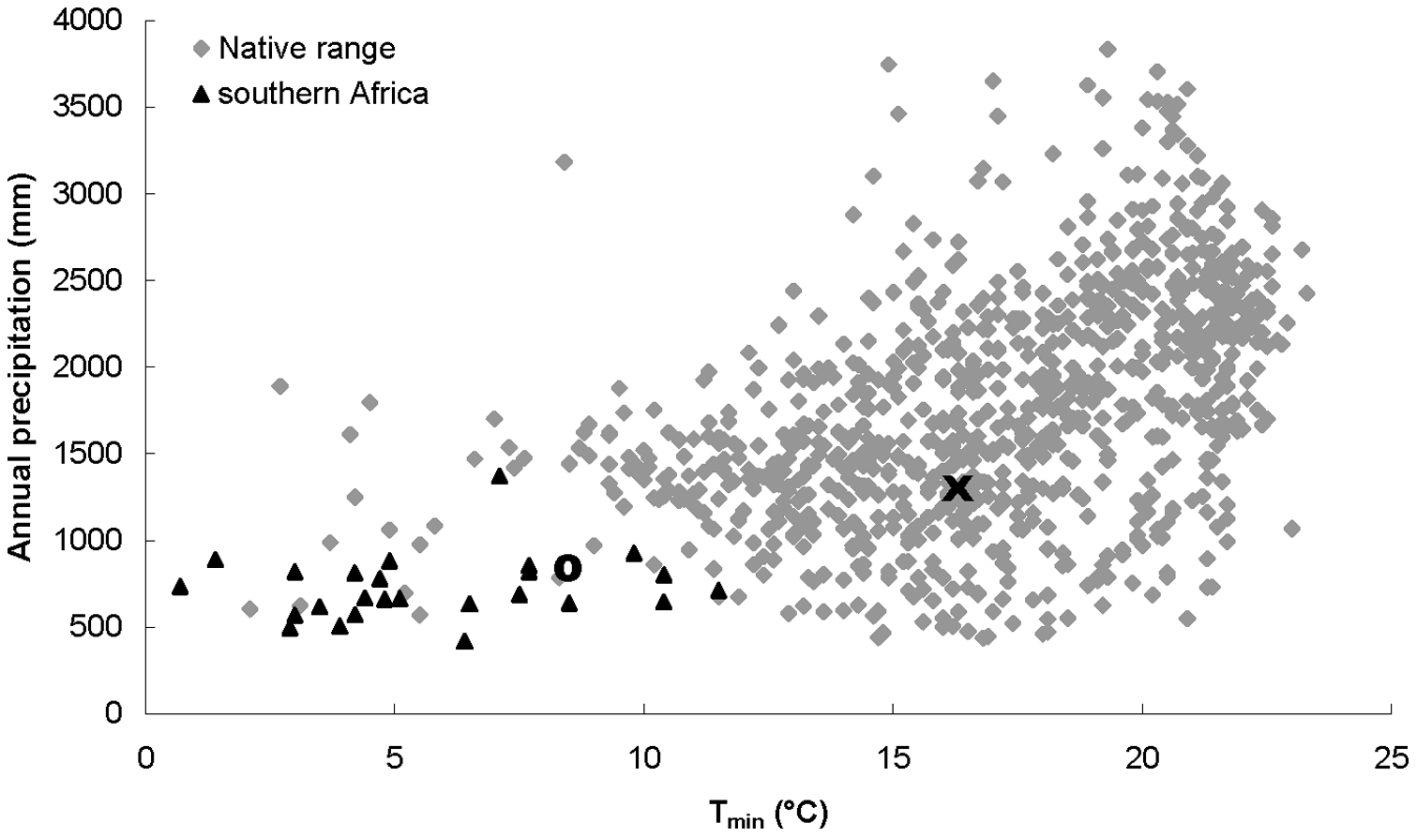
Climatic preference of *Chromolaena odorata* in terms of annual precipitation (y-axis) and minimum temperature of the coldest month (x-axis) in its native range (grey diamonds) and its invasive southern African range (black triangles). The cross indicates the climate from the area where the native population has been collected in Puerto Rico. The circle indicates the climate from the area where the invasive population has been collected in South Africa.

We use the invasion of *Chromolaena odorata* (L.) King and Robinson in mesic savannas in South Africa as a model system to explore factors that determine its current distribution and its potential for future range expansion into more extreme habitats. *Chromolaena odorata* is a widespread neotropical shrub invading a wide variety of ecosystems in the Paleotropics, ranging from tropical rainforests to savannas [6]. The species has been unintentionally introduced in South Africa during the mid-1940 s [12], [13] and is currently invading mesic savannas (>650 mm MAP) in southern Africa that are colder, drier and display a stronger seasonality than most habitats in its native range (Fig. 1) [4], [6], [14]. There is large concern among stakeholders that this nutrient-demanding species will invade drier, semi-arid savannas (<650 mm MAP) at a broad landscape scale as well, where it is currently constricted to riverine habitats [15]. Several studies have suggested that southern African *C. odorata* represents a distinct ecotype, with different climatic preference and morphology [4], [12], [16]. Southern African *C. odorata* plants are less hairy, with smaller leaves and have a more upright growth form than other invasive and native populations. Flowers are white as opposed to pale lilac, with narrow flower heads and bracts with rounded tips [16]. Underground parts have no corm structure which might make them more susceptible for fire [13], [16]. However, this might only be true for smaller plants, as large plants have been shown to be fire-resistant even to high-intensity fires [17]. Biological control programs in South Africa are believed to have failed so far due to the climatic mismatch of invader and biological control agent [14]. The species is intolerant to frost and its range is therefore limited to frost-free areas [13]. Modelling studies predict a great potential for *C. odorata* to expand its non-native distribution based on its native climatic niche [3], [4], [6], notably the savannas in southern and eastern Africa and Australia. In Australia the species is well-contained [3], but in Africa the species is spreading from southern towards eastern Africa [6] and is already present in Mozambique and Tanzania (S. Van Rensburg, pers. comm.). The existing climatic niche models indicate temperature (cold stress) and available moisture (drought stress) to be the main predictors of *C. odorata* distribution [3], [4], [6]. We aim to identify factors that allow *C. odorata* to invade the mesic savannas of southern Africa; habitat which is considered marginal habitat based on the native climatic niche of the species, and explore its potential for further range expansion into more extreme, drier, habitats.

We performed a greenhouse experiment to test the hypotheses whether the invasion in mesic savannas can be explained by (i) increased drought tolerance, or (ii) increased competitive ability of invasive *C. odorata* relative to native *C. odorata*, or (iii) superior competitive interactions of invasive *C. odorata* with the dominant southern African resident grass species *Panicum maximum* (Jacq). Hypotheses (i) and (ii) both invoke genetic differentiation of the South African *C. odorata* population, but (iii) does not necessarily require such a differentiation. Because sub-tropical savannas do not experience frost we focussed on available moisture as the main driver of *C. odorata* expansion. We compared aboveground biomass, biomass allocation, water use efficiency and relative yields of invasive *C. odorata* seedlings under low and high water availability, growing alone or in competition with native *C. odorata* seedlings or with the resident grass *P. maximum*. We expected that invasive *C. odorata* (i) attains a higher biomass and water use efficiency in the dry treatments than native *C. odorata*; (ii) is a stronger competitor than native *C. odorata*; and (iii) is a stronger competitor than the resident grass *P. maximum* under all conditions. We found none of these hypotheses to be true. However, we found that invasive *C. odorata* is superior in light interception, and we discuss how this can explain its invasive success.

## Methods

### Ethics statement

All necessary permits were obtained for the described field studies. The authority who issued the permit for collection in Hluhluwe-iMfolozi Park in South Africa was Ezemvelo KZN Wildlife. In Puerto Rico *C. odorata* was collected on state-owned land, e.g. road verges. No permissions were required for these locations. The field study did not involve endangered or protected species.

### Study site and seed collection

We collected *C. odorata* seeds from three different sites in the species’ non-native range in Hluhluwe–iMfolozi game reserve, South Africa (28° 4′18.52′′ S, 32° 2′23.74′′ E) in November 2004 and from three different sites in its native range in northern Puerto Rico (18° 24′ 40.95′′ N, 66° 34′ 39.74′′ W) in February 2005. The closest sites were at least 10 km apart. We specifically chose Puerto Rico to sample native *C. odorata* populations as previous work has shown that southern African *C. odorata* is likely to have originated from the northern Caribbean [12], [16]. Figure 1 shows the distribution of the native and South African range of *C. odorata* along gradients of minimum temperature and annual precipitation. Distributional data was compiled from several published sources [3], [4], [6], [14] and climatic data was obtained from the WORLDCLIM database (http://www.worldclim.org, version 1.4 (release 3) [18]).

The tall grass species *Panicum maximum* was chosen as a competitor because it co-occurs with *C. odorata* in both ranges and is an important competitor for *C. odorata* in southern Africa. The grass can grow up to 2 m tall, is native to southern Africa and invaded large parts of tropical America. *Panicum maximum* seeds were obtained commercially from McDonalds Seeds, Pietermaritzburg, South Africa. Seeds were germinated in plastic containers on sterile glass beads (*C. odorata*) or sterile soil (*P. maximum*) in the greenhouse (15/25°C, 12 h intervals). For the greenhouse experiment we used seeds from one native and one South African *C. odorata* population, similar to a previous experiment studying the effect of plant-soil interactions on invasion success [19], hereafter called native and invasive *C. odorata,* respectively.

### Field measurements and pilot study

**Figure 2:**
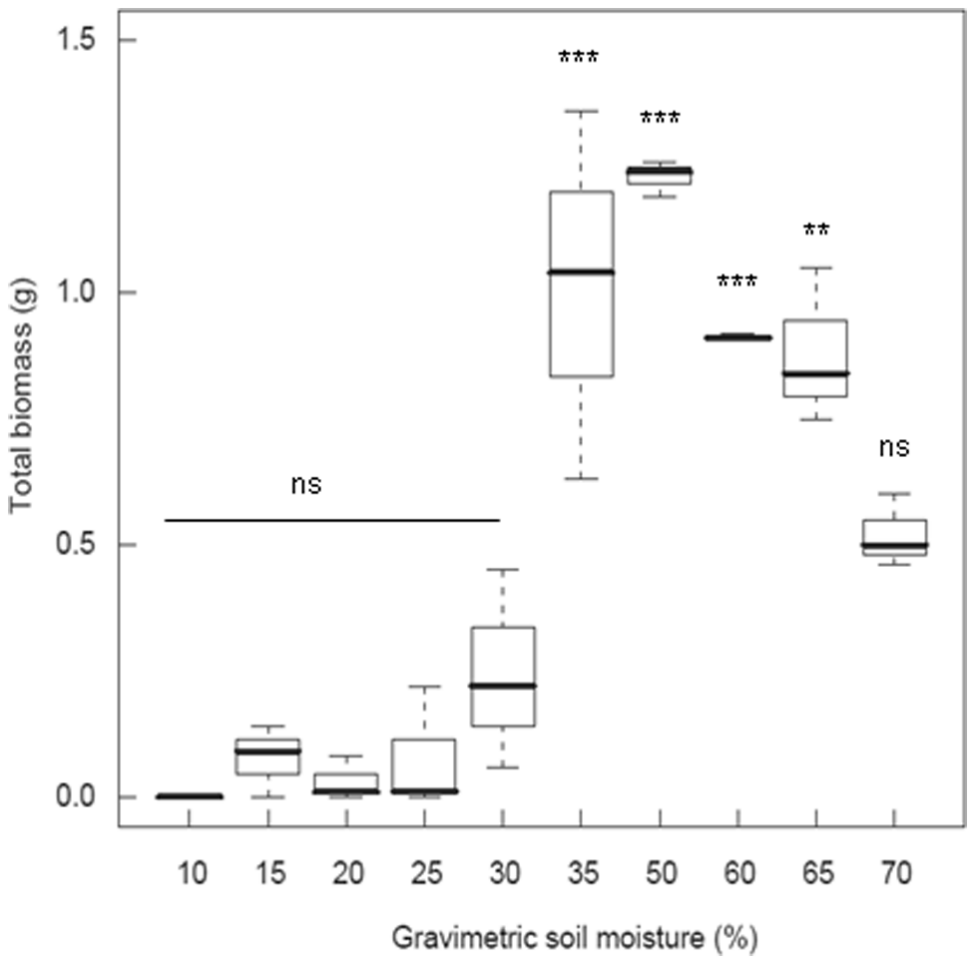
Total biomass of invasive South African *Chromolaena odorata* along an experimental soil moisture gradient. Soil moisture levels are expressed gravimetrically, as percentage of the dry weight of the soil. Results of one-way ANOVA: ***p<0.001, **p<0.01, *p<0.05, ns  =  non-significant.

We performed a greenhouse experiment comparing drought tolerance and competitive ability of native and invasive *C. odorata* under low and high water availability. The levels of the water availability treatment were determined based on field measurements in South Africa and a pilot study measuring the growth response of invasive *C. odorata* along a soil moisture gradient. Soil moisture measurements were performed on three sites (∼700 mm MAP) in Hluhluwe-iMfolozi game reserve with similar soil characteristics. The average field capacity (*i.e.* soil moisture 2–3 days after rain) was 24% (±3%) and the average wet season soil moisture 28% (±6%), dropping to 11% (±7%) in the dry season. The pilot study showed that below 35% soil moisture plant growth was significantly reduced (Fig. 2) and below 30% soil moisture seedlings experienced high mortality. Therefore, we set the dry treatment at 30% soil moisture, which approximates conditions in mesic African savannas. The wet treatment was set at 50% soil moisture, where invasive *C. odorata* showed optimal growth, mimicking conditions in its native range. All soil moisture levels were measured gravimetrically, as percentage of the dry weight of the soil: (wet weight – dry weight) / dry weight. Volumetric soil moisture can be derived from the gravimetric soil moisture by dividing the latter with the specific gravity of field soil (∼1.4 g/cm^3^).

### Experimental design greenhouse study

The greenhouse experiment was performed in Groningen, The Netherlands and set-up in a full-factorial randomised block design with three treatments: water availability (2 levels: high, low), species/population (3 levels: invasive *C. odorata* (Ca), native *C. odorata* (Cp), *P. maximum*) and competition treatment (3 levels, see explained below). After germination the seedlings were transplanted in 3900 ml pots each containing a gamma-sterilised (2.5 kGray) mixture of potting and field soil (1∶1), the latter was collected in a field near the greenhouse. Pots were arranged in blocks in the greenhouse (25/15°C, 12 h intervals) and each block of 18 treatment combinations was replicated five times, resulting in 90 pots. To reduce potential differences in light and temperature within the greenhouse the position of each block was changed every week. Moisture levels were kept constant during the course of the experiment by weighing and watering twice a week. Pots were covered with tin foil to reduce evaporation. The competition treatments consisted of one monoculture treatment, with six individuals per pot of one of the three species/populations, and two mixed cultures with different densities of individuals per pot. In one half of the mixed cultures the total density of plants was kept equal to the density in the monoculture (2×3 individuals per pot), the so-called ‘replacement design’ [20]. In the other half, equal numbers of plants were added to the number in monoculture (2×6 individuals per pot), the so-called ‘additive design’ [21]. To prevent strong nutrient competition, we supplied nutrients in sufficient amounts. Pots were supplied with full strength Hoagland solution once a week [22], beginning two weeks after planting. To meet increasing plant requirements, the amount of Hoagland solution was increased at 2-weeks intervals from 12.5 ml to 25 ml and 50 ml and remained constant after that [23]. Shade cloth was put around each pot to prevent interference between pots. In weeks 5, 7 and 9 we measured light intensity above and below the canopy in the monocultures using a lux meter (LUTRON LX-107), After 10 weeks all plants were harvested. Leaves and stems were separated per plant, dried at 70°C for 24 hours and weighed. Roots were washed, dried and weighed. It was not possible to separate the roots per species in the mixed cultures. We therefore used aboveground biomass rather than total biomass to analyse competitive ability.

### Data analysis

#### Drought tolerance and biomass allocation

We tested for differences between the native and invasive *C. odorata* population in aboveground biomass, water use efficiency and biomass allocation using the monoculture data only (n = 20). Water use efficiency (WUE, g/kg) was calculated as total biomass (g) divided by total amount of water (kg) used during the course of the experiment. To study if differences in biomass allocation could explain the observed patterns we calculated leaf, stem and root weight ratios (LWR, SWR, RWR) for the monocultures as the biomass of each plant part divided by the total biomass. The monoculture data was tested using a mixed-model ANOVA with species and water availability as fixed factors. Block was initially included as a random factor, but because we found no significant effects of or interactions with block, we excluded this factor from the final monoculture analyses. Canopy light interception, *i.e.* the percentage of light intercepted by the plant canopy, was calculated using the ratio between the above- and below-canopy measurements and tested per week using an ANOVA with species and water treatment as fixed factors. Because we found no significant effect of water availability on canopy light interception, we combined data from both water availabilities and tested for differences between species only.

#### Competitive ability

We tested for the effect of all treatments on total aboveground biomass using an ANOVA with competition treatment, species and water treatment as fixed factors. Block was initially included as a random factor, but because we found no significant effects of or interactions with block, we excluded this factor from the final analyses. We analysed these data per species rather then per pot. Because there were two species/populations growing per pot in the competition treatments, the total number of replicates for this analysis was 150 (60×2 plus the 30 monoculture pots with 1 species/population per pot). In a separate analysis we used the data from the replacement design (2×3 individuals per plot) to calculate the strength and direction of competition. We compared the performance of the plants in the mixed cultures relative to the monocultures, the so-called relative yield: RY  =  Y_mix_/Y_mono_ [20], [24]. Relative yields were calculated using aboveground biomass only. Differences in relative yields between the species were tested with univariate ANOVA for each water treatment and competitor pair (n = 10). All data was analysed in R (Version 2.10.0 (2009-10-26)) [25].

## Results

### Drought tolerance and biomass allocation

**Figure 3:**
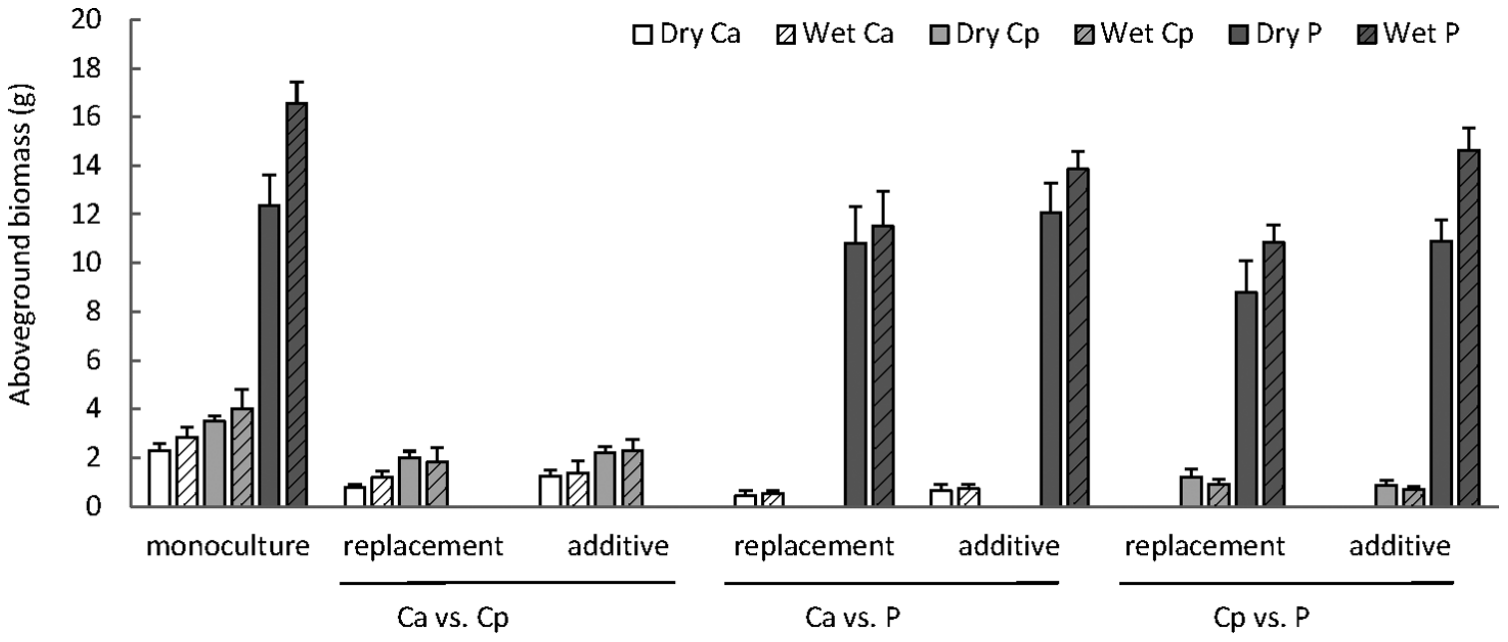
Aboveground biomass of invasive South African *Chromolaena odorata* (Ca, white), native Puerto Rican *C. odorata* (Cp, light grey) and native South African *Panicum maximum* (P, dark grey), with hatched bars indicating the wet treatments. The x-axis shows the competition treaments: monoculture, replacement design (2×3 individual per pot) or additive design (2×6 individual per pot) in intra- and interspecific mixtures.

**Figure 4:**
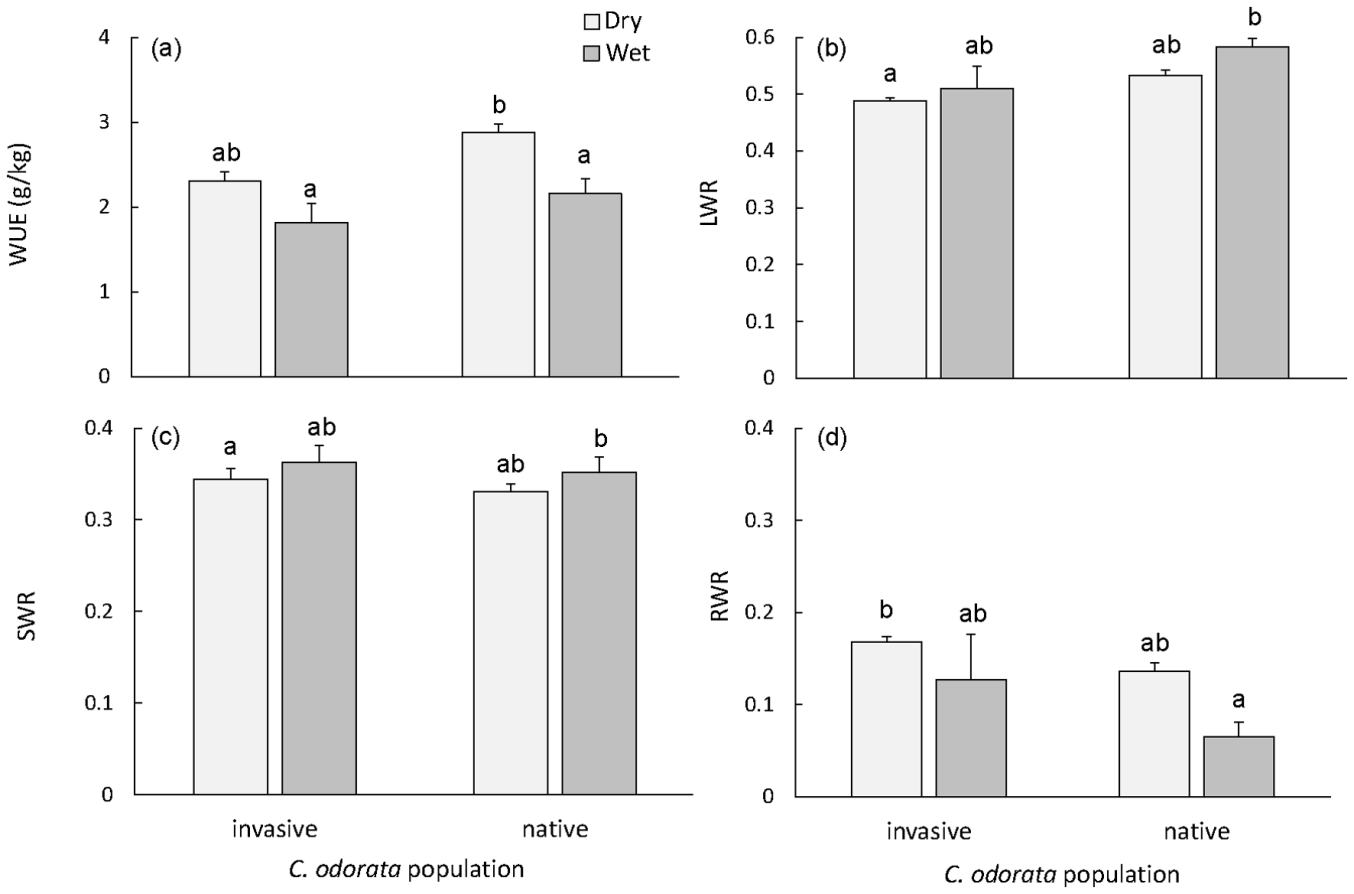
Water use efficiency (WUE) (a), leaf weight ratio (LWR) (b), stem weight ratio (SWR) (c) and root weight ratio (RWR) (d) for invasive *Chromolaena odorata* from South Africa and native *C. odorata* from Puerto Rico for the dry (light grey) and wet (dark grey) treatments. Mean values (+SE) are shown. Data is based on the monoculture treatments only. Letters indicate homogenous groups with p<0.05 (Tukey HSD test). Note that the y-axes have different values.

We did not find evidence that invasive *C. odorata* performed better than native *C. odorata* under drier conditions. Comparing the monoculture data for the *C. odorata* populations only, we found that aboveground biomass did not differ between native and invasive populations (F_1,16_  = 2.6, p = 0.13), irrespective of water treatment (F_1,16_  = 1.2, p = 0.29, data shown in Fig. 3). Water use efficiency was lower under wet conditions than under dry conditions for both populations (F_1,16_  = 14, p<0.01, Fig. 4a), with the native population being more efficient in water use than the invasive population (F_1,16_  = 8.0, p = 0.01).

**Figure 5:**
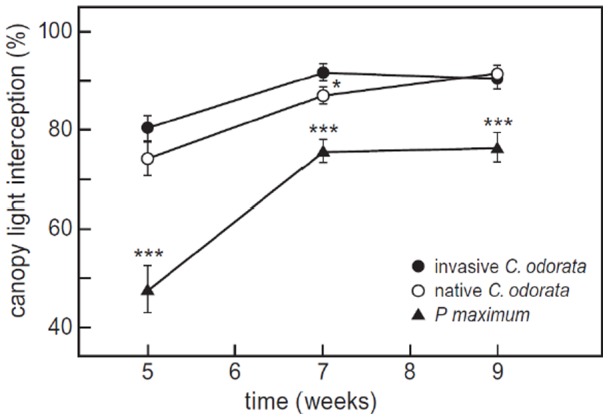
Canopy light interception per species for the monocultures. Solid circles: invasive *Chromolaena odorata* from South Africa; open circles: native *C. odorata* from Puerto Rico; solid triangles: *P. maximum.* Measurements were taken biweekly starting from the 5^th^ week of the experiment. Mean values (±SE) are shown. Results of one-way ANOVA: ***p<0.001, *p<0.05.

Allocation to leaf and stem biomass was higher in the wet treatments than in the dry treatments for both *C. odorata* populations (LWR: F_1,16_  = 5.0, p = 0.04, Fig. 4b, SWR: F_1,16_  = 8.0, p = 0.01, Fig. 4c), whereas allocation to root biomass was lower in the wet than in the dry treatments (RWR: F_1,16_  = 7.7, p = 0.01, Fig. 4d). Allocation to leaf biomass was higher in the native than in the invasive population (LWR: F_1,16_  = 8.6, p<0.01), whereas allocation to stem and root biomass did not differ between populations (SWR: F_1,16_  = 2.2, p = 0.16; RWR: F_1,16_  = 2.3, p = 0.15). The amount of light intercepted by the canopy was highest for invasive *C. odorata* in the beginning of the experiment (Fig. 5). The invasive population was more efficient than the native one in intercepting light in week 7, while at the end of the experiment, in week 9, individuals from both *C. odorata* populations performed equally well, intercepting 90% of the incoming light. Interestingly, both *C. odorata* populations were more efficient in intercepting light than *P. maximum* (F_2,30_  = 13.77, p<0.001, Fig. 5), which only intercepted a maximum of 78% of the available light in week 9.

### Competitive ability

We did not find evidence for increased competitive ability in invasive *C. odorata,* neither in competition with native *C. odorata* nor with *P. maximum*. On the contrary, aboveground biomass of both *C. odorata* populations was significantly lower than that of *Panicum maximum* in all competition treatments (F_2,132_  = 722, p<0.001, Fig. 3). Aboveground biomass for all populations/species was highest in the monocultures and significantly lower in the competition treatments (F_2,132_  = 25.2, p<0.001). Similarly, aboveground biomass was lower in the dry treatments than in the wet treatments (F_1,132_  = 11.4, p<0.001) and this effect was strongest for *P. maximum* (water x species: F_2,132_  = 9.1, p<0.001). *Panicum.maximum* also responded strongest to the density of plants in the competition treatments and attained a higher biomass in the additive design than in the replacement design (competitive design x species: F_4,132_  = 3.3, p = 0.012). The *C. odorata* populations showed no significant differences between additive and replacement designs or between wet and dry treatments.

**Figure 6:**
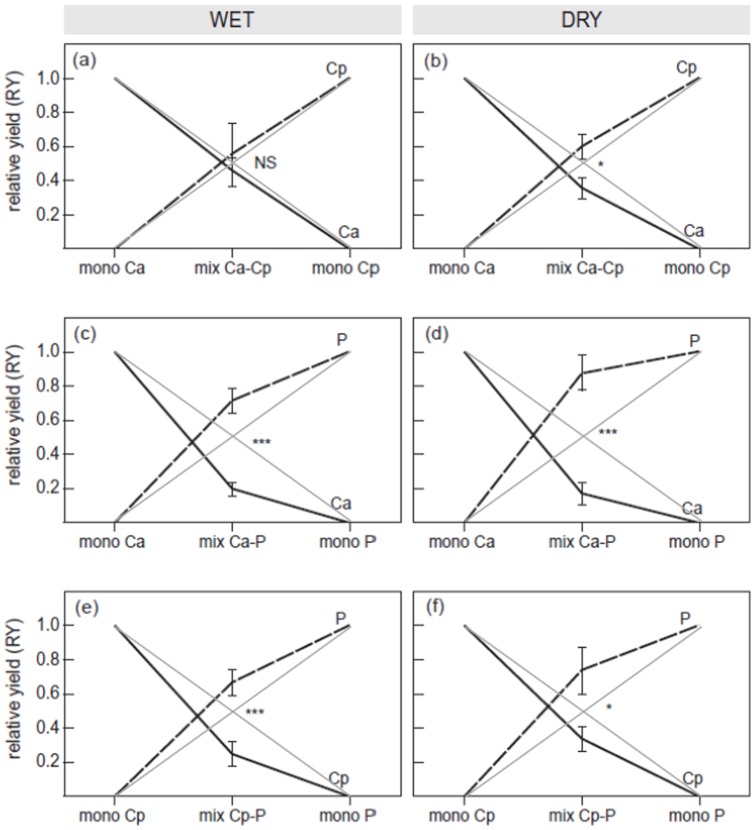
Replacement diagrams showing the effects of the intraspecific competition between native *Chromolaena odorata* (Cp, dashed line) from Puerto Rico and invasive *C. odorata* (Ca, solid line) from South African for the wet (a) and dry (b) treatments; the interspecific competition between invasive *C. odorata* (Ca, solid line) and *P. maximum* (P, dashed line) for the wet (c) and dry (d) treatments and the interspecific competition between native *C. odorata* (Cp, solid line) and *P. maximum* (P, dashed line) for the wet (e) and dry (f) treatments. Relative yields (aboveground biomass in the mixture/aboveground biomass in the monoculture) per species (±SE) are plotted versus the number of plants of the species involved (*e.g.* mono Ca  = 6 invasive *C. odorata* plants grown in monoculture, mix Ca-Cp  = 3 native and 3 invasive *C. odorata* plants grown in mixture, and mono Cp  = 6 native *C. odorata* plants grown in monoculture). If species are not affected by competition, the biomass in the mixed culture must be half of that in monoculture. This situation of equal performance is depicted by the grey lines. Data is based on the replacement design only. Differences in relative yields were tested with one-way ANOVA: ***p<0.001, **p<0.01, *p<0.05, ns  =  non-significant.

To quantify the effect of the competition treatments we calculated relative yields for the replacement design. Relative yield diagrams (Fig. 6) show the effect of competition as the deviation from the point of equal performance ( =  biomass in monoculture divided by 2) and provide a strong visualisation of the strength and direction of competition. This analysis shows again that the invasive population was the inferior competitor, both in competition with native *C. odorata* and with *P. maximum*. The outcome of the competition between both *C. odorata* populations (Fig. 6 a,b) was dependent on the water treatment: in the dry treatment invasive *C. odorata* was the inferior competitor (F_1.8_  = 5.7, p<0.05), while in the wet treatment both populations competed equally well (F_1,8_  = 0.2, p = 0.7). The replacement diagrams for the interspecific competition between the invasive *C. odorata* and *P. maximum* (Fig. 6 c,d) show that *C. odorata* was outcompeted in both water treatments (dry: F_1,8_  = 37.1, p<0.001, wet: F_1,8_  = 43.5, p<0.001). The native *C. odorata* population was also outcompeted by *P. maximum* in both water treatments (dry: F_1,8_  = 6.2, p<0.04, wet: F_1,8_  = 18.6, p = 0.003), but the effects were less strong than for invasive *C. odorata* (Fig. 6e,f).

## Discussion

The invasive South African population of *C. odorata* has not evolved increased drought tolerance under drier conditions that mimic those of mesic savannas. Invasive *C. odorata* did not perform better in the dry treatments, nor did it use water more efficiently than native *C. odorata*. This indicates that no mechanism for more efficient water uptake has evolved in the South African population. Moreover, native *C. odorata* was the stronger competitor under drier conditions compared to the invasive population and the native population responded to these drier conditions with an increase in WUE, a decrease in leaf allocation (LWR) and an increase in root allocation (RWR), which are all strategies to optimise water use. The invasive *C. odorata* population did not respond as strongly, but showed the same increase in RWR in the dry treatments. The lack of water conserving traits in invasive *C. odorata* suggests that it is highly unlikely for invasive South African *C. odorata* to expand to the drier parts of the landscape.

Also, the invasive population of *C. odorata* has not evolved increased competitive ability. Invasive *C. odorata* was not the superior, but the inferior competitor when grown in competition with the common resident grass species *P. maximum* and more so under dry conditions. However, we only used one native and one invasive population. This limits the potential to draw general conclusions on the competitive ability of *C. odorata*. Nevertheless, because previous work has shown low genetic diversity and high morphological homogeneity in South African *C. odorata* [16], we are confident that our results are valuable for the South African *C. odorata* ecotype as a whole.

In the current study we only measured competitive ability in effects on growth reduction, but we recognize that in the long run effects on survival and reproduction may be more important for determining the outcome of competition [26] and the long-term persistence and spread of the invader. Trade-offs exists between optimal growth, survival and reproduction. For example, during range expansion traits associated with dispersal and reproduction are selected for on the expanding front, whereas traits associated with growth and survival show dramatic declines [27]. *Chromolaena odorata* has an enormous seed production of many, yet small, wind-dispersed seeds. A single plant has been reported to produce up to 860.000 seeds, even when conditions are not optimal, resulting in a very high propagule pressure [28]. The trade-off, however, is that small seeds have a slower initial seedling development and lower stress tolerance of the seedlings [29]. This corresponds well with the findings in our experiment that *C. odorata* seedlings are inferior competitors due to slower initial development. We conjecture that the excellent dispersal and reproduction traits in *C. odorata* contribute to its rapid range expansion and success as an invader, despite low competitive ability in the seedling stage.

Moreover, our light measurements show that *C. odorata* is highly effective in intercepting light, which indicates that the species might be a good competitor for light. This is supported by other traits, for example *C. odorata* has a high specific leaf area [30], a high relative growth rate and a high relative investment in stems [19]. Therefore, superior competition for light rather than water might be key to the species’ success, but only if soil nutrients are not limiting and *C. odorata* seedlings are temporarily released from competition in the establishment phase by, for example, disturbance. Previous studies have shown high tolerance of *C. odorata* to disturbances, such as fire or physical damage caused by herbivores or clearing programs [17] and seedling establishment has been shown to increase in the presence of small-scale disturbances of soil and grass layer [31].

Whether or not invasive species evolve increased competitive ability is a controversial issue. The well-studied EICA (Evolution of Increased Competitive Ability) hypothesis states that invasive species can re-allocate resources from defence to growth in the absence of natural enemies [32] and numerous studies supporting and rejecting this hypothesis have been published [33], [34], [35]. A recent analysis [36] shows that the hypothesis might hold for slow-growing species only and not for fast-growing species, such as *C. odorata*. This is further supported by a recent study that did not find evidence for decreased tolerance to herbivory in *C. odorata* [37]. Also, studies have shown that species can be inferior competitors and, at the same time, be invasive, *e.g.* in the presence of disturbance or multiple stable state dynamics [38]. Savanna systems with their inherent environmental variability due to disturbance by herbivores and fire and its multiple stable state dynamics in the form of tree-grass mosaics [39] are perfectly suited to host invasive species that are inferior competitors, at least during some stage in their life cycle. In the current experiment we explored only the seedling stage. However, seedling establishment may be fundamental to the species’ distribution, as previous studies have shown that once established (facilitated by disturbance), *C. odorata* can dominate in the community for over a decade [17], [28].

Predicting the distribution and potential range expansion of invasive species in their non-native ranges is of the utmost importance to mitigate their negative impact. Modeling studies have suggested that the non-native distribution of *C. odorata* did not yet reach its full potential based on its native climatic niche and the species is rapidly expanding especially in southern and eastern Africa [3], [4], [6]. For this reason it is important to better understand the ecological and evolutionary processes that determine its current distribution and whether or not *C. odorata* is likely to show adaptive differentiation to more extreme habitats. A population growing in a marginal habitat can be the starting point for such adaptive differentiation [10], [40]. In our study we did not find evidence for different climatic requirements or increased competitive ability in invasive South African *C. odorata*. Thus, of the scenarios mentioned in the introduction, neither scenario 1 (post-introduction adaptation to the local environment) nor scenario 3 (biotic rather than abiotic constraints in the native range), that both allow expansion of future ranges into habitat that was previously considered unsuitable based on the native range of the species, is likely for *C. odorata*.

Therefore, based on our study scenario 2 (introduction of pre-adapted genotypes and/or genotypes with wide environmental tolerances) seems most likely for *C. odorata*. *Chromolaena odorata* is a species with an extensive native range [6] and a wide environmental tolerance [30], [31] and therefore most likely to grow equally well under different growing conditions, as we showed in the current experiment. Additionally, *C. odorata* has spread from a limited number of introductions [12], [13], is apomictic [28] and shows little genetic variation and high morphological homogeneity in southern Africa [16]. The establishment from a limited number of (pre-adapted) founders that originate from marginal populations, with a different trait spectrum than the native population may allow favourably differentiated traits to be preserved in the population [40]. Moreover, towards their range boundaries, species are thought to have more restricted niches [10] and show more constrained habitat associations [41], which corresponds well with the occurrence of *C. odorata* in (semi-) arid regions being confined to the river valleys [15]. In conclusion, we argue that *C. odorata* is able to invade mesic savannas due to a combination of wide environmental tolerance and pre-adaptation of propagules from marginal native-range habitats and that the specific morphology of the South African ecotype has been attained through founder effects and maintained through asexual reproduction.

Even though in the present study we compared only one native and one invasive population, our work suggests that southern African *C. odorata* has not undergone adaptative differentiation to drier conditions and is therefore unlikely to expand into more extreme, drier, habitats on a regional scale. This implies that the species is currently invading habitats that are at the limits of its climatic tolerance, as determined by available moisture and minimum temperature [4], [6]. Superior light competition, however, might be key in explaining its invasiveness within the confines of its current climatic niche. It is often assumed that marginal populations occur in sub-optimal habitat, and are therefore small, fragmented and vulnerable to stochastic processes [10]. However, our study highlights that species can successfully invade habitats that are at the extreme end of their ranges, even if they are not always superior competitors, and thereby contributes towards a better understanding of range expansion during species invasions.
